# Less Invasive Decompressive Laminectomy and One-Level Lumbar Fusion in the Setting of Interspinous Fixation: A Retrospective Analysis of 15 Patients

**DOI:** 10.7759/cureus.17653

**Published:** 2021-09-01

**Authors:** Hamidreza Aliabadi, Manika S Paul, Mari Kusumi, Barry Chehrazi

**Affiliations:** 1 Neurosurgery, Spine and Neurosurgery Associates, Roseville, USA; 2 General Surgery, Riverside Community Hospital, Riverside, USA; 3 Neurosurgery, Kitasato University Medical Center, Saitama, JPN

**Keywords:** decompressive laminectomy, laminectomy, interspinous fixation, minimally invasive

## Abstract

Lumbar decompressive laminectomy for spinal stenosis can be performed using a less-invasive, unilateral approach with subperiosteal dissection and decompression by undermining the lamina from the ipsilateral to the contralateral side. A unilateral approach to bilateral decompression can be supplemented with interspinous instrumentation and facet fusion, a combined procedure that has not been studied before. The less-invasive technique appears to be as effective for lumbar stenosis as the traditional lumbar laminectomy. It also causes less blood loss and reduced operating time, and so may benefit patients who are elderly, medically frail, or with multiple comorbidities.

Fifteen patients (eight females, seven males) underwent outpatient surgery by the author (HA) using this technique. These patients complained of progressive lower back pain associated with radicular pain exacerbated by prolonged standing or walking with improvement in flexed position of the lumbar spine with decreased walking distance ability. A one-level less-invasive lumbar laminectomy and foraminotomy with facet fusion and interspinous fixation were performed for spinal stenosis in conjunction with a Grade I degenerative spondylolisthesis. These patients all had a single-level facet fusion with bone graft material and local autograft. The approximate surgical time for each patient was between 50 and 80 minutes. The visual analog scale for pain (VAS) score decreased significantly after surgery; patients presented with preoperative VAS scores of 5-10/10 (mean 8.33/10). Postoperative VAS scores were 0-6/10 (mean 2/10), yielding a mean VAS improvement of 76% following surgery. Future analysis should be performed for evaluation of sustained VAS score, Oswestry Disability Index (ODI), Form 36 Health Survey Questionnaire (SF 36), and the Zurich Claudication Questionnaire (ZCQ).

## Introduction

A common pathology associated with Grade I degenerative lumbar spondylolisthesis is neurogenic claudication secondary to spinal stenosis. Neurogenic claudication is a common diagnosis, with an estimated 8% of the adult population affected, and its incidence increasing with aging [1-3]. Pathophysiologically, the combination of facet joint hypertrophy along with annulus fibrosus tears, bulging discs, loss of disc height, and spondylolisthesis with associated ligamentum flavum hypertrophy or calcification may result in spinal stenosis and neurogenic claudication [1]. Therefore, it is commonplace to treat patients with spinal stenosis with a decompressive laminectomy and foraminotomies.

In addition, lumbar spinal fusion has been the standard of care for the treatment of symptomatic degenerative spondylolisthesis. A prospective randomized controlled trial by Herkowitz et al. found that adding fusion was more effective than decompression alone in the treatment of degenerative lumbar spondylolisthesis [4]. Since that time, various fusion constructs to treat lumbar spinal stenosis in the setting of degenerative spondylolisthesis have been considered the gold standard [5], but the possibility remains that a limited spinous process stabilization with facet arthrodesis may be sufficient and may spare patients from some of the complications of traditional pedicle screw fixation-fusion procedures.

Pedicle screw fixation in conjunction with fusion is the most widely used technique for rigid stabilization of the lumbar spine, as it improves arthrodesis rates [5,6]. However, transpedicular fixation is associated with significant complications such as nerve injury, wound infection, and cerebrospinal fluid leakage, and hardware failure such as screw fracture [7]. Furthermore, posterior spinal instrumentation with pedicle screws prolongs operative time and may be associated with significant radiation exposure [8].

To avoid the risks associated with pedicle screw instrumentation, other fixation techniques such as translaminar, transfacet, and interspinous fixation have been developed [8,9]; Wong et al. provide a review of microendoscopic techniques [10]. Interspinous fixation minimizes neurological complications and surgical dissection. It confers shorter operation time than transpedicular fixation and thus may be associated with less post-operative pain and less intraoperative blood loss [9,11] while having a higher reoperation rate under long-term study [12].

The interspinous fixation can also be combined with fusion depending on the patient’s presenting symptoms and imaging. Fusion is particularly indicated in those who display predominantly back pain rather than isolated radicular pain [13]. A caveat with interspinous fixation is that biomechanical studies indicate interspinous fixation devices, compared with pedicle screw fixation, which limits flexion-extension movement but is less effective in limiting axial rotation and lateral bending.

In this study, patients who were older or had multiple comorbidities and presented with back and/or leg pain were treated with interspinous fixation with fusion and a decompressive laminectomy. It was hypothesized that these patients would benefit more from the shorter operation time but were less likely to be affected by the long-term likelihood of reoperation and the reduced limitation of axial rotation and lateral bending.

Just as less-invasive procedures are available for spine stabilization, decompressive laminectomy for central canal stenosis and lateral recess stenosis may be performed with a minimally invasive, unilateral approach for bilateral decompression [14]. Thus, one may focally address the degenerative structures but minimize the destruction of the surrounding normal anatomical structures. These unilateral approaches to lumbar laminectomy have shown benefits typical of minimally invasive surgery, such as decreased blood loss, shorter operating time, shorter hospital stay, decreased postoperative narcotic requirement, decreased rate of infection and cerebrospinal fluid leak, and a decrease in time required for return to work.

Hence, by combining this minimally invasive approach to decompressive laminectomy with interspinous fixation and facet fusion, we are able to obtain equal or better outcomes with less trauma to the normal anatomy [15]. The purpose of this report is to show the effectiveness of our less invasive laminectomy with one-level interspinous fixation and fusion by a retrospective analysis of surgical cases for degenerative spondylolisthesis in the setting of symptomatic spinal stenosis. Decompressive surgery along with a single-level spinous process fixation was performed for each case along with bone graft material and local autograft as an outpatient surgery. The unilateral laminectomy was accomplished in a similar manner to that described in the studies of Thomé et al. and Yaman et al. [15,16], while the interspinous fixation was done similarly to that described in the study by Kim et al. [9]. The full technique is described in the methods section.

North American Spine Society (NASS) 2019 guidelines list a number of indications for using an interspinous stabilization device with fusion [17]; in this study, the patients all had indications for laminectomy and fusion given their spinal stenosis. Therefore, we used interspinous fixation devices in conjunction with fusion. This is one method of achieving the gold standard of direct bony decompressive laminectomy and fusion; it did not rely solely on indirect fusion with an interspinous device (e.g. Vertiflex, Superion, X-stop, or Coflex). As such, our surgical method of posterior bony fusion followed the practice guidelines outlined in the NASS position statements. To our knowledge, while unilateral laminectomy and interspinous fixation have been separately studied, this is the first study of their use in combination for the treatment of lumbar spondylolisthesis.

## Materials and methods

Between September 2012 and January 2017, 15 patients between the ages of 57 and 86 (eight females, seven males) underwent surgery by the author (HA). A one-level less invasive lumbar laminectomy and foraminotomy with facet fusion and interspinous fixation were performed for spinal stenosis in conjunction with a Grade I spondylolisthesis due to degenerative disease. These surgeries were all performed as outpatient surgeries, defined as a facility stay not exceeding 23 hours. 

These patients all had a single-level facet fusion with local autograft and bone graft material (i.e., demineralized bone matrix putty and bone strips). Prior to the operation, patients complained of progressive lower back pain, associated radicular pain, exacerbation of pain by prolonged standing or walking with improvement in the flexed position of the lumbar spine, and decreased walking distance ability. Neurological examinations were performed on all patients before and after surgery. Every patient attempted conservative management prior to surgical therapy including pain medications, physical therapy, anti-inflammatories, anti-spasmodics, and other non-surgical management techniques without significant benefit.

**Figure 1:**
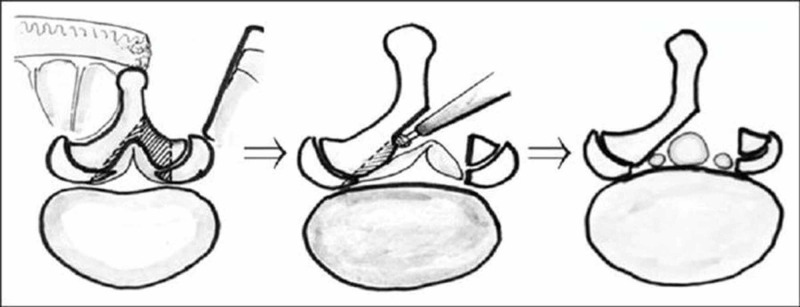
Depiction of the unilateral approach for bilateral decompression. Figure reproduced with permission from Kato, et al. [18].

**Figure 2:**
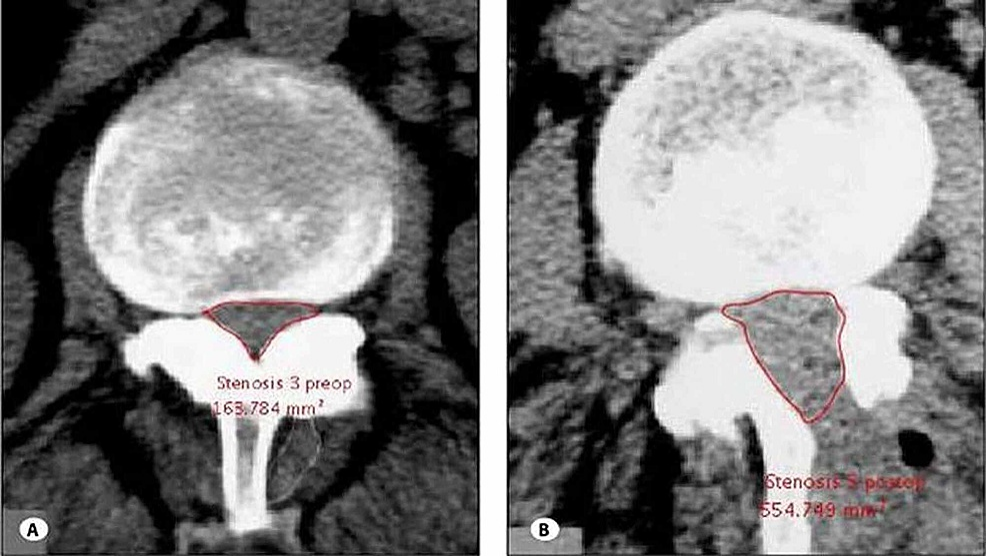
Preoperative (a) and postoperative (b) radiographs showing decompression of the central canal after a unilateral laminectomy and bilateral decompression. Figure reproduced with permission from Yaman et al. [16].

Contralateral fusion was attained via decortication of the facet joint and laminar area, followed by the use of local autograft overlaid by allograft. Fourteen patients had fixation and fusion performed at L4-L5, whereas one patient had surgery at the L3-L4 level. This unilateral approach to bilateral decompression is depicted in Figure 1, while Figure 2 shows a CT comparison of the spinal canal before and after a similar decompression technique.

Surgeries were performed as “outpatient” or “overnight” status. Patient comorbidities and body mass indices were further evaluated (Table 1).

**Table 1:** Patient demographics. HTN: Hypertension; OA: Osteoarthritis; P: Pneumonia; DM: Diabetes; A: Asthma; CAD: Coronary Artery Disease; ICH: Intracerebral Hemorrhage; E: Emphysema; AN: Anemia; HT: Hypothyroidism; CVA: Cerebrovascular Accident; HC: Hepatitis C; PC: Prostate Cancer; COPD: Chronic Obstructive Pulmonary Disease

	Patient	Age	Sex	BMI	Smoker (packs/year)	Comorbidities
1	N.C.	75	F	20.2	2	HTN, HT
2	M.S.	74	F	24.9	0	HTN, OA
3	R.R.	57	F	34.9	0	P, DM, A, CAD
4	C.A.	86	F	25.7	0	HTN, P, AN
5	J.Y.	76	M	30.4	50	P, ICH, CVA
6	R.W.	76	M	29.9	80	HTN, OA, COPD, E, P, CVA
7	L.H.	65	F	35.6	5	OA, P, HP, AN
8	W.B.	82	M	30.3	0	DM, OA, CAD, HTN, AN, CVA, P
9	S.S.	81	F	22.1	5	HTN
10	G.S.	78	M	26.3	0	HTN, HT, DM
11	A.V.	73	F	30.2	0	HTN, OA
12	S.M.	65	F	30.9	0	OA
13	M.H.	63	M	30.3	35	HTN, E, HC
14	T.C.	72	M	30.0	15	PC, OA
15	C.C.	83	M	25.8	34	HTN, CAD, OA

Surgical technique

Patients were placed prone on a Jackson table with a Wilson frame under general endotracheal anesthesia. The Wilson frame was not cranked up. An incision was made with localization using fluoroscopy at the appropriate level of spondylolisthesis and stenosis. A less invasive approach was utilized for a unilateral approach for bilateral decompression of lumbar spinal stenosis with foraminotomies as necessary. 

The average length of our incisions was approximately six centimeters with a posterior midline approach. Following a median lumbar incision, paravertebral muscles were dissected subperiosteally and retracted. The unilateral approach was used for bilateral microscopic decompression. Unilateral medial facetotomy was performed. After decompression was complete, attention was turned to performing a contralateral fusion following decortication. Then interspinous fixation was completed. First, trial sizers were used prior to implantation for correct sizing of the interspinous device. Next, the appropriately sized implant was placed after the removal of the supraspinous and interspinous ligaments. Fixation to the superior transverse process and the inferior spinous process was then carefully and sequentially tightened with a clamp to avoid fracture of the spinous processes. Under fluoroscopy, anteroposterior and lateral x-rays were performed to reveal a good construct in all 15 cases. All fixation devices were tested for stability and strength by manual upward pressure using a Kocher instrument. The implants used were either PrimaLOK™ SP Interspinous Fusion System (OsteoMed Spine, Addison, Texas) or the VIA™ Spinous Process Fixation System (Spineology, St. Paul, Minnesota).

Follow-up X-rays all revealed stable hardware without any worsening spondylolisthesis, with a mean follow-up period of two years.

Clinical assessment

Clinical outcome was assessed subjectively at postsurgical clinical visits as well as by means of a VAS score for assessment of the patient’s lower back and leg pain based on a standard of care procedure. Standard of care x-rays were performed postoperatively to assess construct over time as well.

## Results

**Figure 3:**
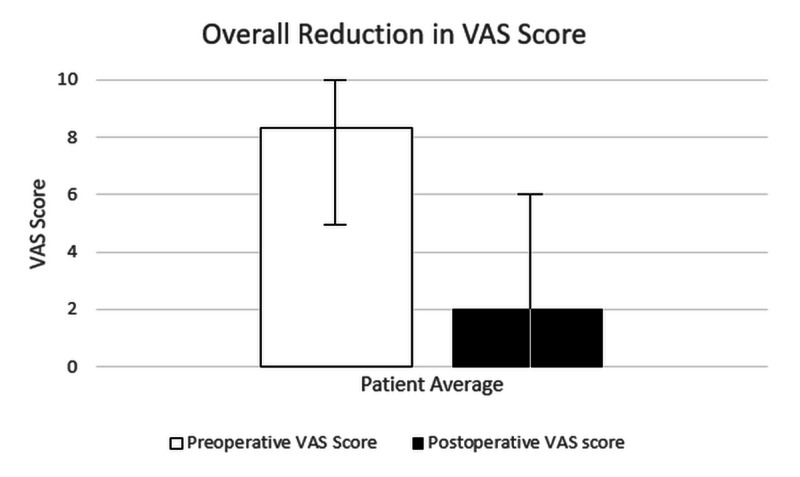
Mean VAS scores for back and leg pain (with range) for 15 patients. VAS: Visual Analogue Scale

There was a significant improvement in the VAS scores for low back and leg pain in these patients postoperatively (Figure 3).

The VAS score decreased significantly after surgery. The patients presented with a VAS of 5-10, with a mean VAS score of 8.33/10. Postoperative VAS scores were 0-6 with a mean postoperative VAS of 2/10, and thus there was a mean VAS improvement of 76% following surgery. This improvement is comparable to that in studies of pedicle screw fixation; one study showed a VAS improvement of 71-73% for posterior-lateral spinal fusion (PSF) in mild degenerative disease [15], with another showing an improvement of 85%, but for a mix of patients with conditions including degenerative spondylolisthesis and spinal stenosis [16].

Further evaluation is needed by objective questionnaires such as the Oswestry Disability Index (ODI) or the Zurich Claudication Questionnaire (ZCQ). However, patient outcomes were excellent based on subjective questioning and healthcare questionnaires. Surgery afforded these patients considerable improvement in their VAS scores for pain compared to preoperative conditions.

## Discussion

The etiology of lumbar stenosis is multifactorial. Causes include ligamentum flavum hypertrophy, osteophyte overgrowth, facet joint hypertrophy, congenital stenosis, disc bulge or herniation, spondylolisthesis, and even in some cases tumors or infections. The 15 post-surgical patients in this study had degenerative lumbar stenosis due to hypertrophy of their ligamentum flavum and facet joints with a Grade I degenerative spondylolisthesis. As a result, these patients likely had a combination of anatomic nerve root compression as well as impaired blood flow to the nerve roots. Traditionally, open bilateral laminectomies have been performed for such stenosis, but now with less invasive approaches, such as the approach used by the surgeon (HA) in these cases, a unilateral approach for bilateral decompression may be undertaken with less resultant surgical trauma or insult. 

The less-invasive approach entails subperiosteal dissection via a unilateral approach for bilateral decompression by undermining the lamina from the ipsilateral to the contralateral side using the plane formed by the ligamentum flavum [19]. This approach appears to be effective in treating lumbar stenosis and is less disruptive to the anatomic structures and paraspinal musculature than the traditional approach. This leads to a lower potential for paraspinal muscle atrophy and long-term spinal instability [15,20]. The traditional approach, on the other hand, includes removal of the spinous process and contralateral lamina.

In earlier literature, this less invasive technique resulted in an excellent visualization and radiographic evidence of decompressed neural elements, and the unilateral approach has achieved similar outcomes with less destruction of native anatomic structures. In 2002, Khoo and Fessler compared this approach to open laminectomy [20]. They found a significant decrease in operative blood loss as well as postoperative narcotic requirements and length of hospital stay. 

Moreover, this less invasive laminectomy method preserves the spinous processes so that spinous process fixation can be combined with fusion. A review of the literature shows patients with preoperative spondylolisthesis have a significantly higher rate (40-100%) of postoperative progression of instability on dynamic x-rays at long-term follow-up [14]. The rate of postoperative progression of spondylolisthesis has been shown to be less in patients who have had arthrodesis and instrumentation [5]. Thus, instrumentation with interspinous fixation may afford the patients less postoperative pain and improved results as well as a shorter surgery time [2,20,21].

Guidelines by the American Association of Neurological Surgeons and the Congress of Neurological Surgeons in 2005 recommended spinal fusion in patients undergoing a lumbar decompression with stenosis and preoperative spondylolisthesis [22]. The concept of spinous process fixation to facilitate arthrodesis has been described in the past, including in NASS guidelines incorporating interspinous fixation and bony decompression [17]. A key advantage of this type of fixation is that it is more readily performed and less invasive than pedicle screw fixation. Unlike pedicle screw fixation, it is not associated with an increased risk to the neural elements.

In the recent era, even though pedicle screw fixation remains the most commonly used technique, alternative fixation techniques, including interspinous fixation devices, have become more popular [15]. Some of the devices developed include spinous process plates, wires, and clamps [2, 23], such as the PrimaLOK SP Interspinous Fusion System and the VIA Spinous Process Fixation System devices used in the current analysis. Also, spinous process fixation can be performed with a shorter operative time than the transpedicular fixation approach with decreased radiation exposure, risk to nerve roots, and risk to anterior vasculature [24].

In this series, we combined the less-invasive unilateral laminectomy approach with the less-traumatic interspinous fixation technique, a combination of techniques that has not been published before. As described, both components of this combined surgery reduce the operative time and various other risk factors in the surgery. All these reductions in risk may make this combined procedure an appealing option in elderly or frail patients or those with multiple comorbidities.

We found that our 15 post-operative patients had a significant decrease in hospital stay. Traditionally, these patients would have required an open laminectomy with pedicle screw fixation and posterolateral fusion, which would have necessitated a longer hospital stay.

The less invasive approach to lumbar laminectomy, which has been briefly described in this paper, has shown benefits such as decreased blood loss, shorter operating time, shorter hospital stay, decreased postoperative narcotic requirement, decreased rate of infection and cerebrospinal fluid leak, and a decrease in time required for return to work. Thus, by combining this less invasive approach with interspinous fixation and facet fusion, we may obtain equal or better outcomes with less trauma and minimal disruption of the normal anatomy.

The question, however, remains as to the long-term efficacy for fusion and stabilization. In previous reports, clinical results have suggested that interspinous fixation for posterior lumbar arthrodesis is effective in promoting arthrodesis. Studies comparing interspinous fixation to pedicle screw fixation have shown generally comparable rates of arthrodesis with around 96% rates of fusion using either method at the one- to two-year mark [25]. Also, interspinous fixation has been effective in stabilizing the spine in biomechanical studies [21]. We have yet to experience any complications in our series of surgeries performed between 2012 and 2017. The other methods such as translaminar or transfacet screw fixation have also been shown to be viable fixation alternatives. However, implantation of these devices requires about the same time and radiologic risk as that of pedicle screw fixation, but with less relative stabilization and also with increased risk to the neural elements. Finally, the longevity of facet screw fixation has also been questioned.

Moreover, studies are needed to compare interspinous devices with other alternative techniques like translaminar facet screws and percutaneous transfacet devices, some of which have demonstrated reduced rates of reoperation, low complication rates, and biomechanical stability [2]. 

Historically, the laminectomies that are performed achieve a 60% success rate in patients, defined as “improved functional outcome” and “patient satisfaction”. The lumbar stenosis trial and spine patient outcomes research trial (SPORT) trial showed similar efficacy with laminectomies for lumbar stenosis. This study shows a benefit in terms of VAS score and functional improvement to patients treated with less invasive lumbar laminectomy and interspinous fixation for Grade I spondylolisthesis. The patients in this study were older individuals with multiple comorbidities or they opted for no pedicle screw fixation hardware. Their average age was 73.7 years. Thus, this procedure may benefit those patients who are medically frail, have multiple comorbidities, or are elderly. Despite their age and comorbidities, the patients did well in the immediate postoperative term while still enjoying a reduction in pain score and improvement in functional ability, which we feel is comparable to and likely much better than with the traditional surgical approaches without the need for blood transfusions, excessive postoperative narcotic requirements or longer hospital stays, and without a higher risk of durotomies or spinal instability.

Limitations of this study include the lack of a control group treated with more traditional approaches for the laminectomy and a relatively small sample size. Furthermore, longer-term follow-up of these patients needs to be completed to determine the long-term effectiveness and complication rates of the procedure as well as possibly the use of the same type of interspinous fixation device. 

Currently, studies of either interspinous fixation or less invasive lumbar laminectomy have followed patients for a few years, but not long enough to determine the rates of long-term complications that are likely to necessitate repeat surgery [19,26]. This data would be of vital importance in determining whether the less invasive or traditional approach causes fewer complications in the long term. Objective measures of pain reduction and functional improvements, such as the sustained VAS score, ODI, SF 36, and ZCQ, would also be important for further analyses.

## Conclusions

We reveal a novel method for one-level less invasive lumbar laminectomy and foraminotomy with facet fusion and interspinous fixation for spinal stenosis in conjunction with Grade I spondylolisthesis due to degenerative disease. VAS score and functional improvement of the patients prove the effectiveness of this surgical approach. For stenosis associated with Grade I spondylolisthesis, this less invasive laminectomy, fixation, and fusion technique would allow for a shorter hospital stay and less postoperative pain than traditional methods.
